# Behavior and Attention Problems in Eight-Year-Old Children with Prenatal Opiate and Poly-Substance Exposure: A Longitudinal Study

**DOI:** 10.1371/journal.pone.0158054

**Published:** 2016-06-23

**Authors:** Egil Nygaard, Kari Slinning, Vibeke Moe, Kristine B. Walhovd

**Affiliations:** 1 Research Group for Lifespan Changes in Brain and Cognition, Department of Psychology, University of Oslo, Oslo, Norway; 2 Center for Child and Adolescent Mental Health, Eastern and Southern Norway (RBUP), Oslo, Norway; 3 Department of Psychology, University of Oslo, Oslo, Norway; 4 Department of Physical Medicine and Rehabilitation, Unit of Neuropsychology, Oslo University Hospital, Oslo, Norway; Shinshu University School of Medicine, JAPAN

## Abstract

Multiple studies have found that children born to mothers with opioid or poly-substance use during pregnancy have more behavior and attention problems and lower cognitive functioning than non-exposed children. The present study aimed to investigate whether behavior and attention problems are more prominent than general cognitive deficits in this risk group and whether the problems wane or increase over time. This prospective longitudinal cross-informant study compared 72 children who were prenatally exposed to heroin and multiple drugs with a group of 58 children without known prenatal risk factors. Group differences in caregivers’ and teachers’ reports of the children’s behavior and attention problems based on the Child Behavior Check List and the ADHD Rating Scale were compared based on group differences in general cognitive functioning at 4 ½ and 8 ½ years of age. Both parent and teacher reports suggest that the exposed group has significantly more problems in several behavioral areas than the comparison group, particularly with regard to attention problems. The preschool teachers had already reported these problems when the children were 4 ½ years old, whereas the caregivers reported these problems mainly when the children were 8 ½ years old. The group differences in behavioral and attentional problems were not significantly greater and some were even significantly smaller than the group differences in general cognitive abilities. These findings suggest that children subject to prenatally drug exposure have increasing problems in multiple areas related to behavior from preschool age to 8 ½ years but that these problems do not seem to be specific; i.e., they are not more severe than the problems with general cognitive abilities found for this group.

## Introduction

Studies on children prenatally exposed to maternal opioid and poly-substance abuse show an increased risk of regulatory problems, such as behavioral and emotional problems [1, 2], aggression, [3, 4], attention deficits [5, 6] and ADHD symptoms [4, 7, 8]. However, because extant findings also indicate that these at-risk groups have other difficulties, including weaker general cognitive abilities [8, 9], little is known about the specificity of the often-emphasized regulatory problems in comparison with general cognitive functioning and the evolution of these problems over time.

Within the illegal drug exposure literature, areas of behavior regulation in infancy that are thought to be affected by prenatal drug exposure have been described as the “Four A’s”: arousal, attention, affect and action [10]. Such dysregulation of arousal, attention, emotions and behaviors is often viewed to be a central aspect of problems with self-regulation [11–13], effortful control [14], emotion regulation [15], executive attention [16] or executive functions [17, 18]. Dysregulation can begin in utero, and it has been proposed to result from a dynamic developmental process in which alterations in the environment modify behavioral expression [1]. Dysregulation in early childhood has been found to be a common precursor of adolescent behavioral and emotional dysregulation symptoms, such as substance use [19] and criminality [20]. The present article investigates the developmental trajectory of behavioral, emotional, social, and attention problems (henceforth referred to as “regulatory problems” in the present article) in opioid- and poly-drug-exposed children who are especially vulnerable to developing such problems.

Although both neuroanatomical studies on humans [21] and experimental studies on animals [22] indicate that prenatal opioid exposure has possible negative consequences for regulatory abilities, questions regarding the extent and nature of these problems remain unanswered. Three of four children born to mothers who used opioids during pregnancy have drug withdrawal symptoms after birth [23] that can be described as neurobehavioral dysregulation [24]. However, whether these infants continue to have drug-related regulatory problems after their abstinences have subsided remains unclear. This uncertainty results from the difficulty of differentiating between prenatal opioid and poly-substance exposure and other prenatal and postnatal contributing factors, the small number and scope of studies on the topic and the discrepancies in the studies’ findings.

Although several studies have reported more regulatory problems among opioid- or poly-drug-exposed children than among non-exposed children [3–7, 25–29], there are many disparities in the findings. For example, there have been discrepancies within studies that use multiple sources of information—that is, regarding the use of both parental vs teacher reports [4], the use of parental reports vs specific neuropsychological tests [7, 26], and the use of specific vs general questionnaires [29]. Furthermore, whereas most studies have found more externalizing behavior or problems with attention and ADHD-related symptoms among children who were exposed to opioids or poly-substances before birth [3–7, 25, 26, 28], findings regarding other aspects of regulatory problems differ. Some studies have found elevated internalizing problems [3], others have not found such problems [4], and others still have found that opioid- or poly-drug-exposed children exhibited a wide array of behavioral and emotional problems [5]. Given the discrepancies across and within studies, there is a need for further investigation concerning the presence of regulatory problems in children born to mothers who used opioids or multiple drugs during pregnancy.

The few studies that investigate both regulatory problems and cognitive abilities in opioid- or poly-drug-exposed children tend to concentrate their analyses and discussion on the most prominent findings, which often are related to attention problems [4–7, 26, 27, 29]. Whereas some studies have not found significant general cognitive problems [3, 7, 26, 27, 30], others have found lower average general cognitive abilities in these risk groups than in comparison groups [5, 6, 8, 9, 29, 31, 32]. If general cognitive abilities are somewhat reduced in drug-exposed children, specific cognitive abilities that are important for regulatory functions might also be negatively affected [33]. Thus, one would expect that the regulatory problems would be partly related to the possible lower general cognitive abilities of opioid- or poly-drug-exposed children. However, to our knowledge, no previous study has investigated whether the levels of regulatory problems in this risk group are related to general cognitive abilities or whether these children have regulatory problems over and above their cognitive deficits. Based on the few studies that have found significant regulatory problems without concordant general cognitive problems in children born to mothers with opioid or poly-drug abuse during pregnancy [7, 26, 27], we would also expect regulatory problems over and above the findings regarding cognitive functioning (e.g., IQ) in the present study.

Most studies on the relationship between prenatal opioid and poly-substance exposure and dysregulation have been cross-sectional studies of young children. Thus, knowledge about how the relation may change over time is lacking. The few longitudinal studies on opioid- and poly-drug-exposed children indicate either a trajectory similar to that of non-exposed children in terms of behavioral and attentional problems [25] and cognitive and psychomotor development [30, 31, 34] or a tendency toward more clearly manifested psychomotor and cognitive difficulties throughout infancy and early childhood [32, 35]. With increasing age, children interact with peers and participate in more complex settings, such as in nursery school and primary school, without close co-regulation from parents. Such environments require specific regulatory abilities, social competencies and cognitive skills. Certain behavioral and emotional challenges may not be recognized as problems until the age at which typical children develop related regulatory skills, such as delayed gratification, waiting for turns, reciprocity in play (turn taking), emotional regulation, tuning out disturbing noises and paying selective attention. [36, 37]. We recently reported that the opioid- and poly-substance-exposed children who are included in the present study had lower cognitive functioning than those in the comparison group and that the discrepancies increased between 3 and 8 years of age [9]. In the present study, we investigated whether these same children also show regulatory problems that increase with age and whether these problems are related to and more extensive than those found for general cognitive functioning.

Children born to mothers with opioid or poly-substance use during pregnancy have several other risk factors beyond the prenatal exposure to substances that should be considered in an evaluation of possible consequences of prenatal substance exposure (e.g., perinatal factors such as lower birth weight and gestational age [38, 39] and the postnatal care environment [7, 30–32]). Thus, the present study controlled for socio-economic status, birth weight and gestational age, and we investigated whether regulatory functioning was related to the number of changes in caregivers and the age at which these changes occurred.

Indications of epigenetic modifications from opioid use, e.g., hyper methylation in the mu-opioid receptor (OPRM1) promoter in opioid-exposed infants [40] and possible trans-generational consequences after preconception exposure [41], have previously been found. Experimental studies of animals and cell cultures have found that prenatal opioid exposure may disturb the central nervous system development (e.g., the neuronal migration and/or the survival rates of brain cells) [42–44] and decrease the length and branch number of the dendrites in the somatosensory cortex [45]. Rodent studies have also found a wide variation of behavioral consequences of prenatal opioid exposure [22]. Thus, we also investigated whether regulatory functioning differs in children with prenatal opioid exposure vs prenatal exposure to other drugs.

The present study thus investigated group differences in attentional, behavioral and emotional problems over time based on caregiver and teacher reports between children with prenatal opiate (heroin) and poly-substance exposure raised in stable and adequate care environments and children without any prenatal drug exposure. Based on the abovementioned studies, we formed three hypotheses: 1) Children who were prenatally exposed to opioids and multiple substances exhibit more caregiver- and teacher-reported problems related to attentional, behavioral and emotional regulation at 8 ½ years than the same-aged children without such prenatal risk factors. 2) Group differences in regulatory problems are specific in that they show significantly larger group differences than general cognitive abilities do. 3) These group differences in regulatory problems increase over time from 4 ½ years until 8 ½ years of age.

## Material and Methods

### Participants

The participants were recruited for a longitudinal study, and originally, 78 infants who were exposed in utero to opiates and other substances (risk group) and 58 infants without any known prenatal or perinatal risk factors were included. The initial sample and results from infancy until age 4 ½ years have previously been described in detail [46, 47]. The children in the risk group were recruited consecutively between 1992 and 1996 from an in-patient clinic for high-risk infants or families with children aged 0–2 years, the Aline Infant and Family Center in Oslo. The majority (77%) of the drug-abusing biological mothers were enrolled in a perinatal risk project located at Oslo university hospital during pregnancy by the second or third trimester, and the rest gave birth at other hospitals outside Oslo. Because one of the aims of the study was to investigate the children’s longitudinal development under conditions of adequate care, the comparison group of non-exposed children was recruited from a non-clinical setting: local maternal and child health centers in Oslo. The present study excluded six children with fetal alcohol syndrome or fetal alcohol spectrum disorder. Thus, the participants in the current study comprised 72 in-utero drug-exposed children (30 girls, 42%) and 58 non-exposed children (23 girls, 40%).

Whereas all of the children in the comparison group lived with their biological families throughout the study period, most of the children in the risk group were either moved to permanent foster homes or adopted before the age of 1 year (*n* = 52 of 72, 72%). The County Social Welfare Board made decisions about child custody after child protection services in Oslo had evaluated the caregiving abilities of the mothers in the substance-exposed group and their motivation to participate in and benefit from a drug- and alcohol-addiction rehabilitation program. Before the Welfare Board’s decision, all of the exposed children lived either with their birth mothers in a family care institution or in a professional short-term foster home. The children moved 1.7 (*SD* = 1.1) times on average before settling with a permanent caregiver. The foster and adoptive parents were strictly screened and selected based on their personal qualities and overall family situation, and they received relevant training and guidance until the child was 3 years old [for more information about the caregiving qualities of the foster and adoptive parents, see 34, 46, 47]. Five children in the risk group still lived with their biological parents at the time of the final assessment. There was a tendency toward higher socioeconomic status among the primary caregivers in the comparison group compared to those in the risk group (Table 1).

**Table 1:** Sample characteristics divided by group.

	Exposed group (*n* = 72)			Comparison group (*n* = 58)			Difference		
	Mean (*SD*)		Range	Mean (*SD*)		Range	95% CI		*p*
							Lower	Upper	
Gestational age (weeks)	38.6	(2.1)	31.0–42.0	40.4	(1.4)	35.0–42.5	-2.5	-1.3	< .001
Birth weight (grams)	3070.2	(643.6)	1160–4380	3707.4	(455.3)	2620–4615	-835.3	-439.1	< .001
Birth head circumference (cm)	34.1	(1.7)	28.0–37.5	35.6	(1.2)	32.0–38.0	-2.1	-1.0	< .001
SES	3.4	(0.9)	1.0–5.0	3.8	(0.9)	1.5–5.0	-0.7	-0.0	.03
Age at first time point (months)^a^	54.1	(8.2)	48–84	50.1	(3.1)	48–60	1.6	6.4	.001
Age at second time point (months)^a^	102.9	(7.5)	80–124	104.5	(5.4)	94–114	-4.1	1.0	.23

Note. Socioeconomic status (SES) was measured on a five-point scale based on both the caregivers’ education level and occupation, with 1 indicating an unskilled worker who only has compulsory education and 5 indicating a caregiver with a profession that requires at least a bachelor’s level education. The differences were tested using Student’s t-test.

^a^ Age was based on the date on which the primary caregivers completed the Child Behavior Check List. There was a high level of missing data for exact age at the first time point. *n*_first time point_ = 56 and 52; *n*_second time point_ = 57 and 48 in the risk and comparison groups, respectively.

Most of the exposed children (*n* = 57 of 72, 79%) were diagnosed with neonatal abstinence syndrome [48]. Ten children were born before gestational week 37 in the risk group, whereas one child was born before gestational week 37 in the comparison group. One child in the comparison group was born at week 35 and had a satisfactory birthweight (3100 grams) and no other signs of prematurity. To avoid sampling bias, this child was not excluded. None of the children in the comparison group and 16 (22%) of the children in the exposed group had low birth weight (< 2500 grams), and the children in the risk group were on average 289.3 grams (95% CI: [106.4, 472.3], *p* = .002, *b*_Z-value_ = 0.45) smaller at birth than those in the comparison group when the analysis controlled for gestational age. See Table 1 for further descriptive information.

Information about prenatal drug exposure in the risk group was gathered from several sources, including both maternal self-reports and the women’s medical and social records [34]. The biological mothers of the children in the risk group used a wide range of drugs [34]. The most common main drug of choice besides tobacco (n = 72, 100%) was opiates (heroin; *n* = 39, 54%), followed by alcohol (*n* = 9, 13%) and benzodiazepines (*n* = 8, 11%). On average, these mothers had used 3.3 different drugs during pregnancy (2 to 6). See the S1 File and S1 Table for further details regarding the mothers’ drug use during pregnancy.

The number of participants varied across time. There were no significant differences between the participants at 8 ½ years (*n* = 104) and those who did not complete the assessment at 8 ½ years (*n* = 26) in terms of gender, drug exposure vs non-exposure, opiate exposure, neonatal abstinence syndrome, gestational age, birth weight, head circumference at birth, caregiver socioeconomic status, age at first or second assessment, or cognitive functioning at 1, 2, 3 or 4 ½ years of age (S2 Table). However, at 8 ½ years, more exposed participants than exposed non-participants had moved to permanent foster or adoptive homes before the age of 1 year, 84% vs 33%, respectively; χ^2^ (1) = 15.8, *p* < .001, *OR* = 10.7, 95% CI [2.9, 38.7].

### Procedure

The researchers used the substance-exposed group’s assessment results as the basis for reports and clinical suggestions when such reports were needed. Thus, the assessments were not blind, and efforts were made to adhere to strict standardized testing procedures. The cognitive assessments at 4 ½ years were conducted by two of the coauthors, whereas the assessments at 8 ½ years were conducted by three clinical psychology graduate students in their final year of study and were supervised by these coauthors (Slinning and Moe). Written informed consent was obtained from the children’s current primary caregivers at both points in time. The study was approved by the Norwegian Social Science Data Services, ref 200200857.

### Measures

The Child Behavior Check List/4-18 version (CBCL) [49] was completed by the child’s primary caregiver when the children were approximately 4 ½ and 8 ½ years old (see Table 1 for the precise ages). The preschool teachers completed the Teacher’s Report Form (TRF) [50] when the children were approximately 4 ½ years old, and the school teachers completed this form when the children were approximately 8 ½ years old. The researchers did not inform the teachers about the children’s background. The CBCL and TRF are among the best cross-culturally validated standardized questionnaire tools for examining behavioral problems in children [51, 52]; e.g., the CBCL/4-18 has a reported one-week test-retest reliability of .82 to .95; a two-year stability of .39 to .87; and good content, construct and criterion-related validity [49, 50]. The CBCL/4-18 and the TRF include 120 statements that describe eight aspects of emotional, behavioral, social and attention problems. The statements can also be categorized into internalizing and externalizing behaviors. Internalizing behaviors include items from three distinct dimensions: withdrawal, somatic complaints and anxiety/depression. Externalizing behaviors include items from two distinct dimensions: aggressive behavior and delinquent behavior. The results for the dimension of thought problems are not presented because of a floor effect in young children. The dimensions of social problems and attention problems are not included in the internalizing or externalizing behavior subcategories and are thus presented separately. There are three response options for the statements: “Not true” (0), “Somewhat or sometimes true” (1), and “Very true or often true” (2). To show the full range of variation, the results are presented as raw scores [49].

The ADHD Rating Scale [53] was completed by the primary caregiver when the child was approximately 4 ½ and 8 ½ years old, by the preschool teacher when the child was approximately 4 ½ years old, and by the school teacher when the child was approximately 8 ½ years old. The ADHD Rating Scale has been reported to have high internal consistency (.94 to .96), high two- to four-week test-retest reliability (.94 to .96), moderate interrater reliability between parents and teachers (.53), and high criterion validity (.63 to .90) [53]. The questionnaire includes 14 questions related to ADHD symptoms. The questions have four response alternatives: “Not at all” (0), “A little” (1), “Pretty much” (2) and “Very much” (3). The responses are summed to create a total score (possible range: 0–42). Cut-offs of eight or more symptoms with a score of 2 or 3 for girls and of 10 or more symptoms with a score of 2 or 3 for boys were used to estimate the proportion of participants with ADHD-related symptoms [54].

The McCarthy Scales of Children’s Abilities [55] and the Wechsler Intelligence Scale for Children–Revised (WISC-R) [56] were used to measure general cognitive functioning when the children were approximately 4 ½ and 8 ½ years old, respectively. The raw scores from 15 subtests on McCarthy were converted to General Cognitive Index scores (expected mean = 100, SD = 16) according to U.S. norms. The raw scores on the WISC-R, which were based on ten different subtests, were converted to a total IQ score (expected mean = 100, *SD* = 15) based on Norwegian norms [57]. The freedom from distractibility factor on the WISC-R was calculated as the sum of the standardized scores for the arithmetic, digit span and coding subtests [58].

### Statistics

The scores were converted to Z-scores (*M* = 0, *SD* = 1) at each time point based on the present sample. This standardization of the effect sizes makes it possible to compare the group differences over time and across the different measurements. Because comparable studies are lacking, it is difficult to determine what constitutes a large effect size. However, we used Cohen’s definition of small (0.2 *SD*), medium (0.5 *SD*) and large (0.8 *SD*) effect sizes [59].

Bivariate analyses of group differences were conducted using Student’s t-test for approximately normally distributed data and the Mann-Whitney U test for data that were not normally distributed (i.e., the CBCL and TRF scores). Mixed effects analyses were used for the multiple linear analyses of group differences. For the variables of interest (group and time), the differences between running the analysis with complete cases and running the analysis with multiple imputed data were small. Because of the known problems of running models with interaction effects on multiple imputed cases [60, 61], the multiple analyses used complete cases. Thus, no imputation of missing data was performed for either the bivariate or multiple analyses. All of the multiple analyses controlled for gender, relative age at the time of assessment, socioeconomic status, gestational age and birth weight (see S1 File for more information about the efforts to control for perinatal factors). The models examining group differences over time included the two-way interaction terms of group*time, group*type of measure (regulatory problem vs cognitive functioning) and time*type of measure, in addition to the three-way interaction term of group*time*type of measure. Therefore, all of the research questions regarding each regulatory problem over time were analyzed simultaneously. To avoid the risk of Type I errors related to multiple analyses, the final p-values were adjusted ad hoc, as suggested by Hochberg and Benjamini [62].

A significance level of .05 was used for all of the analyses, and most of the analyses were run using IBM SPSS statistics version 22. The statistical program R version 3.1.2 with the nlme, lme4, mi and foreign packages was used to control whether the mixed-effects models with complete cases and multiply imputed data provided similar results and to adjust the final p-values for multiple testing.

## Results

### Group Differences at 8 ½ Years of Age

The risk group obtained significantly higher problem scores than the children in the comparison group for aspects of the CBCL and TRF, except for teacher-reported social problems (Table 2). Twelve (21%) of the children in the risk group vs one child (2%) in the comparison group obtained scores that were at or above the 95^th^ percentile on the CBCL total problem scale. The dimension with the most significant group difference was attention problems, for which the caregivers’ reports showed large effect sizes: 14 (25%) children in the risk group, compared with one (2%) in the comparison group, were at or above the 95^th^ percentile on the CBCL attention problems scale. According to the teachers’ responses (TRF), seven (13%) of the children in the risk group, compared with no children in the comparison group, scored at or above the 95^th^ percentile on the total problem scale, and four (7%), compared with one child in the comparison group, scored at or above the 95^th^ percentile on the attention problems scale. Group differences in both externalizing and attention problems reported by the caregivers and the teachers, in addition to social problems reported by the caregivers, were significant after age, gender, socioeconomic status, gestational age and birth weight in addition to multiple analyses were controlled for.

**Table 2:** Attention, behavioral and emotional problems and general cognitive functions for the drug-exposed and comparison groups at 8 ½ years of age.

	Exposed group			Comparison group			Sign. test of diff.			
	*n*	Mean	(*SD*)	*n*	Mean	(*SD*)	Mean difference (Z-value)	95% CI (Z-value)	Bivariate *p*-value^a^	Multiple *p*-value^b^
CBCL Internalizing (caregiver)^c^	57	7.4	6.8	47	4.1	4.3	0.55	0.17, 0.93	.004*	.08
CBCL Externalizing (caregiver)^c^	57	11.3	10.7	47	4.8	6.4	0.68	0.31, 1.05	.001*	.05*
CBCL Social problems (caregiver)^c^	57	2.6	2.8	47	0.9	1.6	0.70	0.33, 1.07	.001*	.02*
CBCL Attention problems (caregiver)^c^	57	5.1	4.2	47	1.7	2.3	0.88	0.52, 1.23	< .001*	.005*
TRF Internalizing (teacher)^c^	60	6.6	7.0	42	3.9	6.5	0.40	-0.00, 0.80	.004*	.09
TRF Externalizing (teacher)^c^	60	9.7	12.0	42	4.2	6.5	0.54	0.14, 0.93	.01*	.05*
TRF Social problems (teacher)^c^	60	2.6	3.5	42	1.3	2.9	0.37	-0.03, 0.77	.06	.25
TRF Attention problems (teacher)^c^	60	9.2	8.5	42	4.2	4.9	0.66	0.27, 1.04	.003*	.01*
ADHD Rating Scale (caregiver)^c^	56	15.0	11.4	46	5.8	5.1	0.91	0.56, 1.26	< .001*	.004*
ADHD Rating Scale (teacher)^c^	52	12.8	10.4	38	6.2	7.0	0.68	0.27, 1.08	.003*	.003*
WISC-R: Total IQ score	55	97.9	16.0	48	116.1	14.2	-1.03	-1.37, -0.70	< .001*	.002*
WISC-R: Freedom from distractibility^c^	55	27.3	6.3	48	33.5	5.6	-0.93	-1.27, -0.58	< .001*	.002*

Note.

^a^ The confidence intervals were calculated using Student’s t-test, whereas the p-values were calculated with the Mann-Whitney U test and adjusted post hoc for 12 multiple tests [62].

^b^ The multiple p-values were calculated using multiple linear regression (mixed-effects models), in which the models included age at the time of assessment, gender, socioeconomic status, gestational age and birth weight as the control variables. The p-values were adjusted post hoc for 12 multiple tests [62]. The multiple analyses were based on complete cases, with *n*_CBCL_ = 57 and 47 for the risk and comparison groups, respectively; *n*_TRF_ = 54 (risk group) and 42 (comparison); *n*_ADHD Rating Scale caregivers_ = 56 (risk group) and 42 (comparison); *n*_ADHD Rating Scale school_ = 50 (risk group) and 38 (comparison); and *n*_WISC-R_ = 55 (risk group) and 48 (comparison).

^c^ Total raw score.

CBCL = Child Behavior Check List; TRF = Teacher Report Form; WISC-R = Wechsler Intelligence Scale for Children–Revised.

* Significant (*p* ≤ .05) prior to post hoc adjustment for multiple tests.

Similar group differences were found for the ADHD Rating Scale. The caregivers’ reports indicated that 14 (25%) of the children in the risk group had scores that were indicative of ADHD problems, and the teachers’ reports indicated that 9 (17%) of the children in the risk group had scores suggesting ADHD-related problems. However, neither the caregivers nor the teachers reported scores above the established ADHD Rating Scale cut-off for any of the non-drug-exposed children [54]. Thus, the risk group had more ADHD-related problems than the comparison group based on the ADHD Rating Scales completed by both the caregivers and the teachers with large and medium effect sizes, respectively (Table 2).

The children in the risk group had lower scores on the freedom from distractibility factor of the WISC-R than the non-drug-exposed group. Large differences between the two groups were also found on the WISC-R total scale of general cognitive functioning. These medium-to-large group differences on the ADHD Rating Scale and the WISC-R were still significant after age, gender, socioeconomic status, gestational age and birth weight, as well as multiple analyses, were controlled for (Table 2). See S3 Table for a complete correlation matrix of all regulatory problems and cognitive functions for the drug-exposed and comparison groups at 8 ½ years of age.

### Regulatory Problems Relative to General Cognitive Functioning

Multiple mixed-effects models were used to analyze the differences in regulatory problems between the risk group and the comparison group at both assessments and to determine whether these group differences were larger than the group differences in general cognitive functioning (Table 3).

**Table 3:** Changes over time in the group differences in attention, behavioral and emotional problems and general cognitive functions.

	Group difference at 4 ½ years				Group difference at 8 ½ years				Significance of differences in the changes over time between groups^a^
	Diff. mean	95% CI	Multiple *p*-value^a^	Relative to cognitive functioning, multiple *p*-value	Diff. mean	95% CI	Multiple *p*-value^a^	Relative to cognitive functioning, multiple *p*-value	Multiple *p*-value
CBCL Internalizing	0.05	-0.31, 0.42	.61	.04*	0.55	0.17, 0.93	.07	.11*	.17*
CBCL Externalizing	0.20	-0.12, 0.53	.84	.08*	0.68	0.31, 1.05	.02*	.19	.17*
CBCL Social problems	0.37	0.03, 0.71	.41	.37	0.70	0.33, 1.07	.02*	.19	.31
CBCL Attention problems	0.43	0.11, 0.76	.41	.44	0.88	0.52, 1.23	.001*	.56	.17*
TRF Internalizing	0.62	0.27, 0.97	.03*	.88	0.38	-0.02, 0.78	.23	.05*	.39
TRF Externalizing	0.76	0.42, 1.11	.004*	.88	0.55	0.15, 0.95	.06*	.11*	.39
TRF Social problems	0.68	0.33, 1.03	.02*	.88	0.38	-0.03, 0.78	.27	.05*	.33
TRF Attention problems	0.91	0.58, 1.24	< .001*	.57	0.66	0.27, 1.05	.02*	.19	.36
ADHD Rating Scale (caregivers)	0.53	(0.19, 0.88)	.10	.57	0.87	(0.50, 1.24)	.001*	.58	.31
ADHD Rating Scale (teacher)	0.90	(0.56, 1.24)	< .001*	.57	0.66	(0.26, 1.06)	.03*	.19	.36
General cognitive functioning	-0.73	(-0.39, -1.08)	.04*		-1.03	(-0.70, -1.37)	.001*		.33

Note. The mean group differences are presented as Z-values (*M* = 0, *SD* = 1).

The number of participants differed over time. Only participants with complete data for each time point are included. *n*_CBCL at 4 ½ years_ = 56 (risk group) and 52 (comparison); *n*_CBCL at 8 ½ years_ = 57 (risk group) and 47 (comparison); *n*_TRF at 4 ½_ = 61 (risk group) and 54 (comparison); *n*_TRF at 8 ½_ = 54 (risk group) and 42 (comparison); *n*_ADHD Rating scale caregivers 4 ½ years_ = 56 (risk group) and 52 (comparison group); *n*_ADHD Rating scale caregivers 8 ½ years_ = 56 (risk group) and 42 (comparison group); *n*_ADHD Rating scale school 4 ½ years_ = 58 (risk group) and 53 (comparison group); *n*_ADHD Rating scale school 8 ½ years_ = 50 (risk group) and 38 (comparison group); *n*_General cognitive functioning 4 ½ years_ = 55 (risk group) and 49 (comparison group); and *n*_General cognitive functioning 8 ½ years_ = 55 (risk group) and 48 (comparison group). The estimated group differences with a 95% CI were calculated using Student’s t-test. A positive number indicates a higher score (more regulatory problems or better cognitive functioning) in the risk group than in the comparison group.

^a^ The significance of the group differences and the relative group differences (interaction between group and type of measurement (behavior problem vs cognitive functioning)) at each assessment and the changes in group differences over time (interaction between group and time) were analyzed using 11 multiple mixed-effects models that controlled for relative age at the time of assessment, gender, socioeconomic status, gestational age and birth weight. The p-values were adjusted post hoc for 11 tests at 4 ½ years and 8 ½ years and for changes over time [62].

CBCL = Child Behavior Check List; TRF = Teacher Report Form.

* Significant (*p* ≤ .05) prior to post hoc adjustment for multiple tests.

In contrast to what was expected, group differences in regulatory problems, as measured by the CBCL, TRF and ADHD Rating Scale, were generally similar to or smaller than the group differences in general cognitive functioning (Table 3). At 4 ½ years, all the group differences in caregivers’ reports, on both the CBCL and the ADHD Rating Scale, went in the direction of smaller group differences in regulatory problems than in cognitive abilities and even significantly smaller differences for internalizing problems. The preschool teachers’ reports at 4 ½ years did not show significantly more group differences for regulatory problems than for general cognitive abilities. At 8 ½ years, all reports by caregivers and teachers showed fewer group differences for regulatory problems than for general cognitive abilities, although these differences were significant only for teachers’ reports regarding internalizing and social problems after all covariates and multiple testing were controlled for (Table 3).

### Changes in Group Differences over Time

The same mixed-effects models used to investigate relative differences in regulatory problems were also used to determine whether group differences waned, persisted, or increased over time (Table 3). As expected, the direction of the group differences indicated that the risk group was disadvantage with respect to all of the measures at all times (Table 3). However, there were large fluctuations in how many more problems the risk group was reported to have and how these differences changed over time.

**Fig 1:**
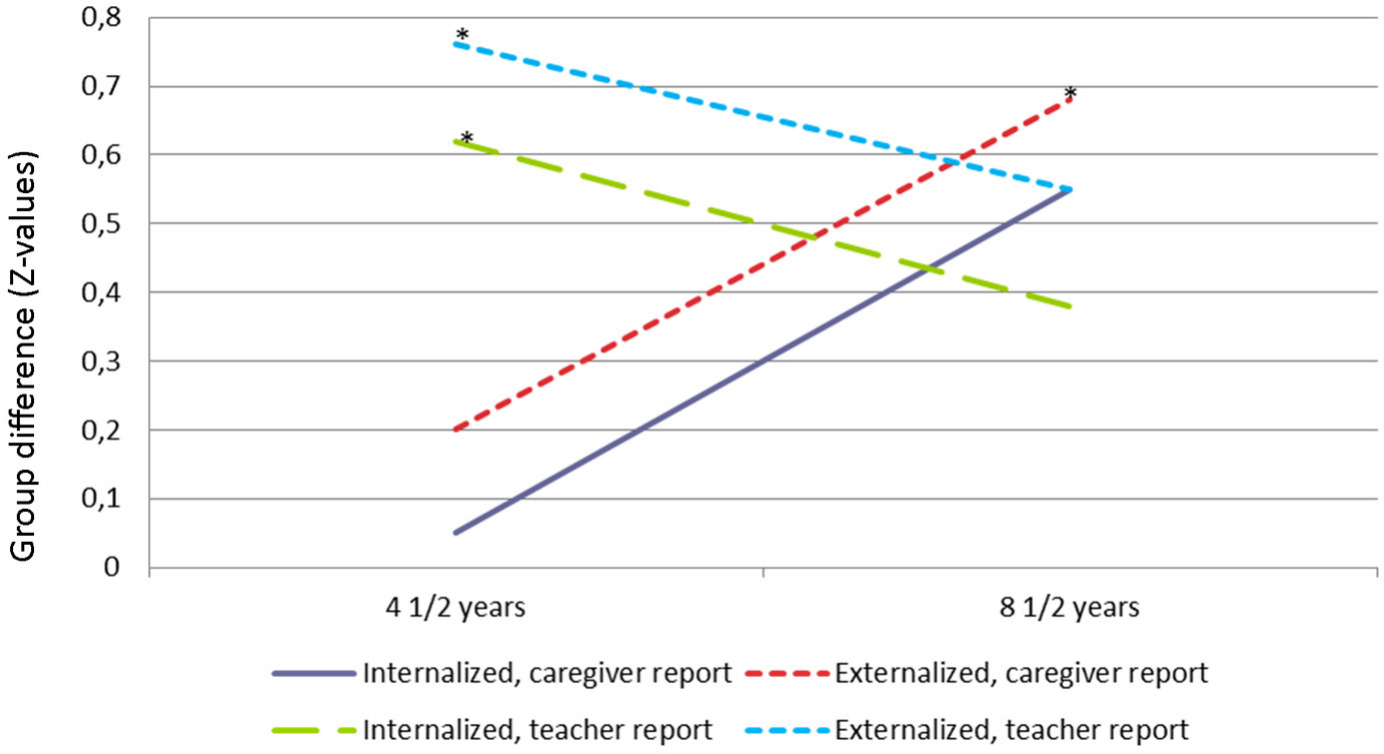
Mean difference between the risk and comparison groups for internalizing and externalizing problems. All of the figures estimate how much higher (in standard deviations) the caregivers’ and teachers’ reports (the CBCL and TRF scores) for the risk group were in comparison with the comparison group across time. * Significant group difference with p ≤ .05 when the analysis controlled for relative age at the time of assessment, gender, socioeconomic status, gestational age and birth weight and was adjusted for multiple analyses.

The smallest group differences in the caregivers’ reports of problems occurred at the 4 ½-year evaluation; none was significant when demographic and perinatal factors were controlled for (Table 3). The greatest group differences in caregiver-reported problems were at the 8 ½-year evaluation (Fig 1). The increases in the between-group differences in the caregivers’ reports of internalizing, externalizing and attention problems between 4 ½ and 8 ½ years were small to medium and were significant when the relative age at the time of assessment, gender, socioeconomic status, gestational age and birth weight were controlled for (Table 3). However, none of these changes was significant when the p-values were adjusted for multiple testing.

There were significant group differences in all of the preschool teachers’ reports when the children were 4 ½ years old (Fig 1 and Table 3). These differences were medium to large and were especially noteworthy for attention problems. The schoolteachers’ reports when the children were 8 ½ years old were similar in terms of the group differences in the preschool teachers’ reports at the previous assessment. However, only the differences in attention problems were significant when covariates and multiple testing were controlled for. There were no significant changes in group differences from 4 ½ to 8 ½ years of age on the reports that used the TRF (Table 3).

The caregivers’ and teachers’ reports on the ADHD Rating Scale were significantly higher for the risk group than for the comparison group at both 4 ½ and 8 ½ years with medium-to-large effect sizes. However, there was no significant change over time (Table 3).

The mixed-effects models included the three-way interaction terms between group, time and type of assessment (regulatory problem vs cognitive functioning) to analyze whether there were any significant (p ≤ .05) changes over time in the group differences in regulatory problems vs cognitive functioning. None of these three-way interaction terms was significant, either before or after multiple testing was controlled for (results not shown). Thus, there were no indications that the group differences in regulatory problems relative to general cognitive functioning changed over time.

### Opiates vs Other Drugs

The sub-sample of children whose mothers used heroin as their main drug of choice (*n* = 39) did not significantly differ from that of children born to mothers with another main drug of choice (*n* = 33) in terms of birth weight, head circumference at birth or caregiver’s socioeconomic status; however, the children of heroin users had a somewhat lower gestational age, *M*_Heroin_ = 38.1, *SD* = 2.2 vs *M*_Other_ = 39.1, *SD* = 1.7; *p* = .03, *b*_Z-value_ = 0.51, 95% CI [0.04, 0.98]. There were no significant bivariate differences between the subsample of children whose mothers used heroin as their main drug of choice and the children born to mothers with another main drug of choice in terms of any caregiver or school report on regulatory problems or cognitive functioning at 4 ½ or 8 ½ years of age.

### Change of Caregiver

Because most of the children born to mothers who used drugs during pregnancy were moved to foster or adoptive homes, there was not sufficient variation within the sample to evaluate whether those moves were related to the children’s regulatory problems. However, there was some variation in how many times the children changed caregivers and at what age they had their final change of caregiver. There were no significant differences in the caregiver or teacher reports of regulatory problems or general cognitive abilities at 4 ½ or 8 ½ years of age between the children with one or zero caregiver changes (*n* = 29) and those with two or more caregiver changes (*n* = 38) when the children who were known to live with their biological mothers at the final assessment were excluded. There were no significant bivariate relationships between the child’s age at the last change of caregiver and any of the caregiver or teacher reports on regulatory problems at any age except for caregiver-reported attention problems at 4 ½ years of age (*r*_*s*_ = .26, *p* = .04), and this relationship was no longer significant when demographical and perinatal factors were controlled for, *b*_Ranked cases_ = 0.04, 95% CI = [-0.02, 0.11], *p* = .16. The age at the last change of caregiver was not significantly related to the test results for cognitive functioning at 4 ½ or at 8 ½ years.

## Discussion

### Group Differences at 8 ½ Years of Age

Our first hypothesis was supported. Children who were prenatally exposed to opioids or multiple substances had more regulatory problems at 8 ½ years of age than children without such exposure. Both the drug-exposed children’s caregivers and their teachers reported a broad range of regulatory problems, particularly a high level of attention problems. These problems were reflected in the informants’ responses on the CBCL, the TRF and the ADHD Rating Scale. High levels of attention problems in children prenatally exposed to opioids or multiple drugs have been reported in earlier studies [4–7, 25–27, 29]. The other regulatory problems that were reported by the caregivers and teachers are also in accordance with previous findings. For example, de Cubas and Field [3] found that school-aged children who had been prenatally exposed to methadone exhibited greater anxiety and aggression problems than non-drug-exposed children. Other studies have reported that drug-exposed children exhibited behavioral problems [25, 28] but not necessarily internalizing problems [4]. However, Davis and Templer [5] found that children who were prenatally exposed to opioids fared worse in almost every psychosocial area, including anxiety and aggression, than children who were not prenatally exposed to drugs but who lived among drug users. Although an earlier study [7] and the present study found that caregivers’ and teachers’ reports yielded the most significant group differences for attention problems and the least for internalizing problems, the drug-exposed children in both studies also seemed to exhibit internalizing problems, such as anxiety and depression, and externalizing behavior problems. Thus, our findings that the children have both internalizing, externalizing, social and attention problems are in accordance with the few previous studies investigating a broad spectrum of regulatory problems.

### Regulatory Problems Relative to General Cognitive Functioning

The second hypothesis was not supported. Our results indicate that the regulatory problems are not more severe than general cognitive problems. Thus, although the reports from caregivers and teachers indicate that prenatally drug-exposed children have more regulatory problems than non-exposed children, these group differences are similar to or even smaller than the group differences in general cognitive functioning.

No previous study has compared the level of regulatory problems with the level of general cognitive abilities in children born to mothers with opiate or poly-substance use, although some discuss or analyze both behavioral and cognitive measures as specific problems [3, 4, 7, 25, 63–65]. However, our finding raises the possibility that the identified regulatory problems may be a reflection of a lower general level of functioning rather than a specific problem for this group. Executive functions are general-purpose control mechanisms that modulate the operation of cognitive sub-processes [18]. Executive functions are thereby theoretically highly related to both regulatory problems and general cognitive functioning [66, 67], and they may thus be an important explanatory link for the present findings. Different aspects of attention are core elements of executive functions [67]. Thus, the findings that attention is one of the most commonly reported regulatory problems among drug-exposed children support the centrality of executive functions for the group differences found in the current study. However, the lack of substantial correlations between regulatory problems and cognitive abilities (S3 Table and S2 File), especially freedom from distractibility [58], makes the results difficult to interpret. Neither the present study nor any previous study is able to disentangle the causal mechanisms by which regulatory problems, executive functions and general cognitive functioning probably interact in transactional processes throughout children’s lives [68].

### Changes in Group Differences over Time

We encountered a few discrepancies when evaluating our third hypothesis, which suggested that the differences in regulatory problems between the two groups would increase over time. The caregivers’ reports suggested that the problems increase rather than decrease. There were no significant group differences on the 4 ½ years CBCL caregiver reports, when we controlled for confounding variables and multiple analyses. However, there was a tendency toward increased group differences in internalizing and externalizing behavior, particularly attention problems, in the caregivers’ reports between 4 ½ and 8 ½ years of age (Fig 1 and Table 3). Although the increase in the caregiver-reported ADHD Rating Scale scores between the 4 ½-year and 8 ½-year follow-ups was not significant, the trend was similar. There were no such increases in group differences over time in the teachers’ reports. Rather, there were already significant group differences in internalizing and externalizing behavior, social problems and attention problems, with a particularly large effect size for attention problems, in the preschool teachers’ reports when the children were 4 ½ years old. Thus, there seemed to be cross-informant agreement about which types of problems characterized the risk group. However, the preschool teachers, who have a much greater basis for comparing different children’s functioning than parents in general have, observed the problems earlier than the caregivers did.

The finding of a possible increase in regulatory difficulties over time is similar to the increase found in group differences in general cognitive functioning between the ages of 4 and 8 years in the present sample [9] and to similar increases reported in other studies [32, 35]. Some longitudinal studies have found similar developmental trajectories for drug-exposed and non-exposed children [25, 30, 31, 34]; however, most of those studies investigated children under 4 years of age [30, 31, 34]. Both the risk and comparison group in the study by Crea, Barth [25] comprised children who had been adopted; thus, both groups may have had some vulnerability, and problem behaviors increased up to four years after adoption in both groups before they decreased. Thus, the developmental trajectory of children born to mothers who used opioids or poly-substances during pregnancy may be similar to that of children without such risks until the children reach 4 years of age. The developmental trajectory of these vulnerable children may then be more negative than that of children in the comparison group for both cognitive and regulatory functioning in preschool and at early school ages. The trajectory may be similar to that of other children once the at-risk children pass this vulnerable transitional age.

Although the sample size and lack of variation reduced the possibility of analyzing realistic and complex models, the increase in regulatory problems may be understood in terms of a transactional model [68] of cumulative risks [69]. Individual vulnerabilities and environmental factors influence each other over time in a continuous, dynamic, transactional way. Thus, biomedical factors that are present at birth, such as genetic makeup, the possible neurological effects of prenatal drug exposure and other related prenatal risk factors (bad nutrition, stress), may interact with later environmental factors, such as quality of parental caregiving, to influence the child’s abilities to regulate his or her emotions and behaviors. The theoretical perspective of such transactional processes is in line with a more recent understanding of biological mechanisms of behavioral changes, which indicates that prenatal risk factors may have epigenetic consequences that are moderated or mediated by the children’s postnatal experiences [70]. A child’s natural developmental course also places increasing demands on the child. As children grow, their behavior and cognition become more complex and thus demand the use of more complex executive functions [36]. These vulnerable children may have benefited during their early years from having a stable placement with specially selected foster or adoptive parents. When they enter kindergarten and school, however, they face a more complex and less protective social environment, and their vulnerability is challenged. The difference in demands across situations may also explain why the preschool teachers reported regulatory problems at 4 ½ years, whereas the caregivers reported these problems later. Although the teachers did not receive information about the children’s background during the study, the preschool teachers may have had more knowledge about the children’s background than the schoolteachers because when the children were in preschool, less time had passed since their placement in a foster or adoptive home. Thus, there may have been observer bias differences between the preschool teachers and schoolteachers that primed the preschool teachers to expect and report more problems among the children born to mothers who used drugs during pregnancy.

### Opiates vs Other Drugs

Although studies on both human infants and rodents have found that possible epigenetic modifications are associated with opioid use [40, 41] and although experimental animal and cell culture studies have found disturbances in the development of the central nervous system [42–45] and behavioral consequences after prenatal opioid exposure [22], there have been very few studies of children born to mothers with opioid use during pregnancy. The present study did not find any significant differences in regulatory problems at any time between the children born to mothers who used heroin as their main drug of choice and the children whose mothers used other drugs; thus, the possible negative effect of opiate and poly-substance exposure may be similar to that of other types of prenatal poly-substance exposure. Heroin use may have been underreported by the mothers, who were facing an evaluation by authorities regarding whether their children should be moved to another caregiver. Although other drugs, such as alcohol, nicotine and benzodiazepines, can cause similar symptoms, neonatal abstinence syndrome is thought to occur mainly after prenatal opioid exposure [24] and not from exposure to marijuana or cocaine, for example [2]. Thus, the present study’s finding that 79% of the risk group experienced neonatal withdrawal symptoms but that only 61% (S1 Table) of the mothers reported using heroin at any time during pregnancy indicates an underreporting of opioid use. Alcohol is known to be one of the most detrimental substances to a developing fetus [2], and it is known to influence regulatory functioning, such as attention and behavioral problems [71]. Opioid-using mothers often tend to use substances other than alcohol [72]. Thus, the lower alcohol use among mothers with heroin as their main drug (10%) than among the other substance-abusing mothers (58%) may have influenced the group differences in regulatory problems in opposite directions. Whereas the children born to mothers with heroin as their main drug of choice may have been more negatively influenced by heroin, most of them may simultaneously have been protected from the negative effects of prenatal alcohol use.

### Change of Caregiver

Neither the number of times the children changed caregivers nor the age at permanent placement was significantly related to the children’s regulatory problems at any time. The children in the risk group who had the worse starting points were probably moved earliest to alternative care. However, they did not have worse outcomes at 8 ½ years. Thus, the care provided by the permanent foster and adoptive parents may have compensated for part of the children’s less favorable starting point. Most of the children were moved early to permanent foster or adoptive homes. Thus, the present sample may have had too little variation to reveal a relationship between their regulatory problems and caregiver changes. A change in caregivers may not be a reliable measure of the quality of the care, and other measures of care quality may better reflect this potentially important factor. Unfortunately, we did not measure quality of care at the last assessment. A video study of a 20-minute play session between the mother and child was conducted for a subsample of the children (n = 54) at 4 ½ years of age, and the results showed that the caregivers of the children in the risk group provided more help, motivated the children more, were more actively involved and structured the children’s play with a puzzle more than the parents in the comparison group [73]. These interaction tendencies were related to lower concordant self-regulation abilities in the children; thus, they may be interpreted as a form of scaffolding, in which the caregivers adapt their level of support to the children’s needs [74].

### Limitations

The sample size was small in comparison with the number of analyses that were completed, which increases the risk that the results were caused by random fluctuations. We compensated for this problem by adjusting the p-values for multiple analyses. However, this method may have increased the risk of hiding true findings. The low number of participants also minimized the possibility for us to control for additional covariate factors, such as different combinations of drugs and family factors. Gestational age and birth weight can be related to prenatal drug exposure and prenatal maternal stress [75] and smoking [76]; thus, the multiple analyses that controlled for these perinatal factors may have underestimated the effect of prenatal drug exposure. Another methodological factor that may have contributed to an underestimation of the group differences is the exclusion of six children with symptoms of fetal alcohol spectrum disorder. Some of these symptoms could be viewed to have resulted from prenatal opioid or poly-drug exposure.

Our results are based on primary caregivers’ and teachers’ reports. It is difficult for caregivers and teachers to correctly assess children’s regulatory problems and separate their own experiences from children’s behavior and functioning. However, this discrepancy is most common for internalizing problems [77] and is less common for externalizing or attention problems, which were the most widely reported regulatory problems in the present study. Cross-informant discrepancies are also often interpreted as differences across situations rather than as a sign of low validity [49]. The reports of group differences across informants and the findings of clear group differences on the WISC-R also indicate that the group differences in the present study were real.

The mixed-effects model’s assumption of normally distributed data was not fulfilled. However, because of the difficulties of nonparametric bootstrapping, linear models were used [60], and the standard errors and reported p-values may be too low. These problems do not indicate any systematic errors in the estimates.

The present study cannot isolate the effects of drug exposure in utero, and the differences between the groups may result from a number of risk factors in addition to prenatal drug exposure. First, the children in the risk group may have hereditary risks that were passed down from their biological parents. The present study did not assess the cognitive functioning of the biological mothers, and there was no valid information available about mental illnesses among the biological parents. Second, the children in the risk group may have had detrimental experiences with their biological parents before they moved in with their final caregivers. Common risk factors that co-occur with maternal opioid and poly-drug use, which may also influence the care situation, include parental mental health problems, family violence, low socioeconomic status, poverty, and a lack of social support. However, most of the children (74%) in the present study moved to their final caregivers before they turned 1 year old. The early age of placement and intense follow-up by the perinatal risk project team before the placement should have minimized the effect of a detrimental early environment. Third, the care that the adoptive and foster parents provided may have differed from the care that the biological parents provided. However, the adoptive and foster parents in the present study were stable and permanent, were specially selected and trained to care for at-risk children and had a relatively higher socioeconomic status than what is common in Norway [34, 78]. A study of foster parents in Norway also found that although foster children may have more cognitive deficits than those in a comparison group, there were no significant differences in their attachment style or in the foster parents’ ability to care for the children [79, 80]. Thus, there are indications that most of the children who were placed with foster and adoptive parents were raised in normal, stable, caring family environments.

The birth-mothers in the risk-group used multiple drugs during pregnancy. The majority reported heroin as their main drug of choice (other than tobacco), and the outcomes for this subgroup of heroin-exposed children did not differ from the results for the rest of the risk group. Studies of meconium from the offspring of mothers on methadone have reported poly-drug use patterns that are similar to those of opioid-dependent mothers who do not use methadone [72]. Thus, although it is impossible to differentiate the consequences of the various drugs the children were exposed to prenatally in the present sample, exposure in our sample may be comparable to that in other groups of children of opioid-dependent mothers.

The comparison group was a convenience sample, and it may not be representative of the Norwegian population. The comparison group’s mean IQ of above 100 could have resulted from both the relatively high socioeconomic status of the a comparison group and the Flynn effect owing to the approximately 15 years between the Norwegian standardization of the WISC-R and our use of the test in the present study [81]. The comparison group’s mean CBCL results (Table 2) are similar to those of a large, normative Norwegian sample [82], which indicates that the between-group differences in the caregivers’ reports did not result from the lack of representativeness of the comparison group.

### Conclusions

According to our results, children with prenatal opioid and poly-substance exposure who mainly were raised by foster or adoptive parents have significantly more caregiver- and teacher-reported regulatory problems than children in a non-exposed comparison group. In addition to attention problems, drug-exposed children exhibit more internalized emotional regulation problems, such as anxiety and depression, and externalized aggressive behavior. However, the regulatory problems do not seem to be specific, as similar or even larger group differences were found in general cognitive functioning. According to the caregivers’ reports, the regulatory problems seem to increase rather than decrease as the children enter more complex social situations, such as preschool and school, whereas preschool teachers may have already reported such problems in preschool. Thus, the regulatory problems and changes therein may be situation specific. Given both the considerable variation within the group and the tendency to develop more problems over time, these children need long-term assessment and follow up, at least into school age, in order to ensure that the most vulnerable children receive the necessary help. Further longitudinal research is needed to clarify the relative contributions of different co-occurring risk and protective factors over time, e.g., genetic vulnerability, the combination of drugs used during pregnancy, relative birth weight, parental vs alternative care and the quality thereof, situation-specific vulnerabilities, and the interventions provided in preschool and school.

## Supporting Information

S1 File Additional Information about the Methods. (DOCX)

S2 File Relationship between Regulatory Problems and Cognitive Functioning. (DOCX)

S1 Table Biological mothers’ use of substances during pregnancy (*n* = 72). (DOCX)

S2 Table Differences between the participating (i.e., those whose parents completed the CBCL) and the non-participating children at 8 ½ years. (DOCX)

S3 Table Correlation matrix of regulatory problems and cognitive functions for the drug-exposed and comparison groups at 8 ½ years of age. (DOCX)
