# Supplementary material for: Behavior and Attention Problems in Eight-Year-Old Children with Prenatal Opiate and Poly-Substance Exposure: A Longitudinal Study
Source: PLoS One. 2016 Jun 23;11(6):e0158054. doi: 10.1371/journal.pone.0158054 (PMC4918960; doi:10.1371/journal.pone.0158054)
Supplement: S1 File — (DOCX) [file pone.0158054.s001.docx]

# Supplementary Material

# Appendix S1 Additional Information about the Methods

## Additional Information about the Participants

The study included mainly children who were placed in stable foster or adoptive homes from an early age. As is common in clinical samples, there is a lack of concurrent information about the care environment. Thus, the study focused more on the changes that occurred than on what predicted these changes.

Information about the mother’s use of drugs during pregnancy was based on the mother’s medical and social records and from interviews with the mothers. As is common for such clinical samples, regular toxicological test results during pregnancy for the mothers were not available. Because of heavy substance abuse and other related factors, it was difficult to obtain reliable information from the mothers about the amount, frequency and timing of drug use, so we have no reliable measures of such details concerning their drug use during pregnancy. For these reasons, we only include what is likely to be the most reliable information: the women’s main drug of choice during pregnancy and the other drugs they used during pregnancy. On average, the mothers who used drugs during pregnancy used 3.3 different drugs, including tobacco (Supplementary material Table 1S). The subsample of 39 mothers who reported that heroin was their main drug of choice besides tobacco used 3.4 different drugs on average. For this subgroup, most frequently reported drugs that were used during pregnancy were tobacco (100%), benzodiazepines (56.4%), cannabis (33.3%) and amphetamines (20.5%). Only four (10.3%) women in this subgroup reported having used alcohol during pregnancy, as compared to 19 (57.6%) among the mothers with other main drug of choice than heroin (chi-square = 18.4, p < .001).

Because of the number of participants, the combination of drugs used, and the lack of information about the amount and timing of drug use, it is difficult to determine the effects of each substance. Smoking could be a confounding factor, but it could not be controlled for because all of the mothers of the drug-exposed children smoked during pregnancy. However, other studies have found that smoking cannot fully explain reduced fetal growth in prenatally opioid-exposed children [1].

## Additional Information about Statistical Procedures

All of the multiple regression analyses controlled for birth weight and gestational age. The perinatal factors of low birth weight and early gestation age have been found to be predictors of later cognitive abilities [2] and brain volume, even variations in birth weight within the normal range have predictive value [3]. Birth weight and gestational age can be seen as indicators of the child’s prenatal environment; for example, they are related to maternal stress during pregnancy [4] and maternal use of substances, such as tobacco [5], during pregnancy. However, it is also probable that maternal use of opioids and multiple substances will influence birth weight and gestation age [6]. Thus, even though we controlled for perinatal factors to try to avoid other prenatal factors’ influence on cognitive abilities, it is possible that the inclusion of birth weight and gestational age as covariates may underestimate the effect of prenatal opioid and poly-substance exposure. Gestational age and birth weight are highly related. Because the effect of the perinatal factors per se is not the topic of the present article, both perinatal factors were included as covariates to control for their possible direct effects.

## References for Appendix S1

1. Mactier H, Shipton D, Dryden C, Tappin DM. Reduced fetal growth in methadone-maintained pregnancies is not fully explained by smoking or socio-economic deprivation. Addiction. 2014;109(3):482-8. doi: 10.1111/Add.12400.

2. Leitner Y, Fattal-Valevski A, Geva R, Bassan H, Posner E, Kutai M, et al. Six-year follow-up of children with intrauterine growth retardation: Long-term, prospective study. J Child Neurol. 2000;15(12):781-6. doi: 10.1177/088307380001501202.

3. Walhovd KB, Fjell AM, Brown TT, Kuperman JM, Chung Y, Hagler DJ, Jr., et al. Long-term influence of normal variation in neonatal characteristics on human brain development. Proc Natl Acad Sci U S A. 2012;109(49):20089-94. doi: 10.1073/pnas.1208180109.

4. Monk C, Spicer J, Champagne FA. Linking prenatal maternal adversity to developmental outcomes in infants: The role of epigenetic pathways. Dev Psychopathol. 2012;24(4):1361-76. doi: 10.1017/S0954579412000764.

5. England LJ, Kendrick JS, Wilson HG, Merritt RK, Gargiullo PM, Zahniser SC. Effects of smoking reduction during pregnancy on the birth weight of term infants. Am J Epidemiol. 2001;154(8):694-701. doi: 10.1093/aje/154.8.694.

6. Creanga AA, Sabel JC, Ko JY, Wasserman CR, Shapiro-Mendoza CK, Taylor P, et al. Maternal drug use and its effect on neonates: a population-based study in Washington State. Obstet Gynecol Clin North Am. 2012;119(5):924-33. doi: 10.1097/AOG.0b013e31824ea276.
