# Supplementary material for: Behavior and Attention Problems in Eight-Year-Old Children with Prenatal Opiate and Poly-Substance Exposure: A Longitudinal Study
Source: PLoS One. 2016 Jun 23;11(6):e0158054. doi: 10.1371/journal.pone.0158054 (PMC4918960; doi:10.1371/journal.pone.0158054)
Supplement: S2 File — (DOCX) [file pone.0158054.s002.docx]

# Relationship between Regulatory Problems and Cognitive Functioning

A bivariate and partial correlation matrix of all of the assessments of regulatory problems and cognitive abilities for the drug-exposed group and the comparison group at approximately 8 ½ years of age is presented in Supplementary Table S3. General cognitive abilities were negatively correlated with most measures of regulatory problems, although most of the correlations were non-significant. None of the correlations between general cognitive abilities and regulatory problems were significant within the comparison group. However, while the general cognitive abilities of the risk group were significantly negatively correlated with the caregivers’ reports of the children’s externalizing, social and attention problems, they were not correlated with any of the teachers’ reports. The correlations with the caregivers’ responses, however, were rather small (statistically significant partial correlations between -.30 and -.44).

Both the classic marshmallow study by Mischel, Shoda [1] and many later studies [for a review, see, e.g., 2] found a relationship between regulatory functions and general cognitive abilities. However, as in the present study, the correlations were normally small. One probable main reason for the low correlations, in both the present study and others, is that whereas cognitive abilities are assessed using neuropsychological tests, the broad range of regulatory problems is usually assessed with questionnaires. This is in accordance with, for example, a study that found through latent-variable analysis that questionnaires and tests of effortful control seemed to measure different underlying latent variables [3] and a review study that found that 68 of 286 correlations between performance-based measures and ratings of executive control were statistically non-significant with a median correlation of .19 [4]. This is also supported by the very high correlations between questionnaires from the same respondent (e.g., a partial correlation of .87 between the two teacher reports concerning attention problems in the risk group, compared with the correlations of -.25 and -.20 between the test results for freedom from distractibility and the two questionnaire-based measures (Supplementary Table S3).

## References for Appendix S2

1. Mischel W, Shoda Y, Peake PK. The Nature of Adolescent Competencies Predicted by Preschool Delay of Gratification. J Pers Soc Psychol. 1988;54(4):687-96. doi: Doi 10.1037//0022-3514.54.4.687.

2. Nisbett RE, Aronson J, Blair C, Dickens W, Flynn J, Halpern DF, et al. Intelligence: new findings and theoretical developments. Am Psychol. 2012;67(2):130-59. doi: 10.1037/a0026699.

3. Samyn V, Roeyers H, Bijttebier P, Rosseel Y, Wiersema JR. Assessing effortful control in typical and atypical development: Are questionnaires and neuropsychological measures interchangeable? A latent-variable analysis. Res Dev Disabil. 2015;36:587-99. doi: 10.1016/j.ridd.2014.10.018.

4. Toplak ME, West RF, Stanovich KE. Practitioner Review: Do performance-based measures and ratings of executive function assess the same construct? J Child Psychol Psyc. 2013;54(2):131-43. doi: 10.1111/jcpp.12001.
