# Supplementary material for: Behavior and Attention Problems in Eight-Year-Old Children with Prenatal Opiate and Poly-Substance Exposure: A Longitudinal Study
Source: PLoS One. 2016 Jun 23;11(6):e0158054. doi: 10.1371/journal.pone.0158054 (PMC4918960; doi:10.1371/journal.pone.0158054)
Supplement: S1 Table — (DOCX) [file pone.0158054.s003.docx]

Supplementary Table S1

Biological mothers’ use of substances during pregnancy (*n* = 72)

|  | Main drug of choice^a^ | | Reported use of drug | |
| --- | --- | --- | --- | --- |
|  | *n* | % | *n* | % |
| Tobacco | 72 | 100.0 | 72 | 100.0 |
| Heroin | 39 | 54.2 | 44 | 61.1 |
| Benzodiazepines | 9 | 12.5 | 36 | 50.0 |
| Alcohol | 8 | 11.1 | 23 | 31.9 |
| Psychopharmaca^b^ | 6 | 8.3 | 10 | 13.9 |
| Cannabis | 4 | 5.6 | 22 | 30.6 |
| Amphetamines | 4 | 5.6 | 14 | 19.4 |
| Cocaine | 1 | 1.4 | 5 | 6.9 |
| Barbiturates | 0 | 0 | 4 | 5.6 |
| Ecstasy | 0 | 0 | 2 | 2.8 |
| Other | 1 | 1.4 | 5 | 6.9 |

^a^ Main drug of choice besides tobacco.

^b^ Antipsychotic medications
