# Supplementary material for: Behavior and Attention Problems in Eight-Year-Old Children with Prenatal Opiate and Poly-Substance Exposure: A Longitudinal Study
Source: PLoS One. 2016 Jun 23;11(6):e0158054. doi: 10.1371/journal.pone.0158054 (PMC4918960; doi:10.1371/journal.pone.0158054)
Supplement: S2 Table — (DOCX) [file pone.0158054.s004.docx]

Supplementary Table S2

Differences between the participating (i.e., those whose parents completed the CBCL) and the non-participating children at 8 ½ years

|  | Non-participating children (*n* = 26) | | Participating children (*n* = 104) | | Difference | | |
| --- | --- | --- | --- | --- | --- | --- | --- |
|  | Mean (SD) or  *n* (%) | | Mean (SD) or  *n* (%) | | 95 % CI | | *P* |
|  |  |  |  |  | Lower | Upper |  |
| Group (risk group) | 15 | (58%) | 57 | (55%) |  |  | .79 |
| Gender (boys) | 15 | (58%) | 62 | (60%) |  |  | .86 |
| Stable care (moved before 1 year)^a^ | 5 | (33%) | 48 | (84%) |  |  | < .001 |
| Mother’s main drug (opiates)^a^ | 9 | (60%) | 30 | (53%) |  |  | .61 |
| Neonatal abstinence syndrome (yes)^a^ | 11 | (73%) | 46 | (81%) |  |  | .53 |
| Gestational age (weeks) | 39.4 | (1.6) | 39.4 | (2.1) | -0.8 | 0.9 | .92 |
| Birth weight (grams) | 3255.4 | (601.2) | 3379.3 | (660.4) | -157.8 | 405.6 | .39 |
| Birth head circumference (cm) | 34.7 | (1.5) | 34.8 | (1.7) | -0.6 | 0.8 | .78 |
| Socioeconomic status | 3.4 | (1.0) | 3.7 | (0.9) | -0.1 | 0.7 | .15 |
| Cognitive score at 1 year^b^ | 96.2 | (12.3) | 95.1 | (12.6) | -6.7 | 4.4 | .68 |
| Cognitive score at 2 years^c^ | 100.2 | (17.4) | 96.7 | (14.1) | -10.4 | 3.3 | .30 |
| Cognitive score at 3 years^d^ | 99.3 | (14.0) | 98.5 | (11.5) | -6.3 | 4.5 | .75 |
| Cognitive score at 4 ½ years^e^ | 106.5 | (15.3) | 107.7 | (15.1) | -5.6 | 8.0 | .73 |
| Age at first CBCL time point (months)^f^ | 25.9 | (3.4) | 26.0 | (3.0) | -1.7 | 1.7 | .99 |
| Age at second CBCL time point (months)^g^ | 53.0 | (7.4) | 52.0 | (6.4) | -4.2 | 2.2 | .54 |

Note. Student’s t-test was used to analyze the differences in means, and the Pearson chi-square test was used to analyze the differences in the grouped variables. The children’s general cognitive abilities were assessed with the Bayley-II Mental Development Index at 1, 2 and 3 years of age and with the McCarthy General Cognitive Index at 4 ½ years.

^a^ *n* = 15 non-participants + 57 participants who were prenatally exposed to drugs

^b^ *n*_1 year_ = 25 non-participants and 99 participants at 8 ½ years

^c^ *n*_2 years_ = 23 non-participants and 97 participants at 8 ½ years

^d^ *n*_3 years_ = 24 non-participants and 102 participants at 8 ½ years

^e^ *n*_4 ½ years_ = 24 non-participants and 101 participants at 8 ½ years

^f^ *n*_first assessment_ = 16 non-participants and 75 participants at 8 ½ years

^g^ *n*_second assessment_ = 20 non-participants and 88 participants at 8 ½ years

CBCL = Child Behavior Check List
