# Supplementary material for: Behavior and Attention Problems in Eight-Year-Old Children with Prenatal Opiate and Poly-Substance Exposure: A Longitudinal Study
Source: PLoS One. 2016 Jun 23;11(6):e0158054. doi: 10.1371/journal.pone.0158054 (PMC4918960; doi:10.1371/journal.pone.0158054)
Supplement: S3 Table — (DOCX) [file pone.0158054.s005.docx]

Supplementary Table S3

Correlation matrix of regulatory problems and cognitive functions for the drug-exposed and comparison groups at 8 ½ years of age

|  | **1.** | **2.** | **3.** | **4.** | **5.** | **6.** | **7.** | **8.** | **9.** | **10.** | **11.** | **12.** |
| --- | --- | --- | --- | --- | --- | --- | --- | --- | --- | --- | --- | --- |
| 1. CBCL Internalizing (caregiver) |  | **.58 /** *.46* | **.58 / .54** | **.70 / .77** | **.51 /** *.40* | .12 / .07 | **.54 /** *.44* | .20 / .21 | **.46 /** *.40* | .08 / .03 | -.24 / -.19 | -.24 / -.18 |
| 1. CBCL Externalizing (caregiver) | *.50 /* **.71** |  | **.62 / .61** | **.67 / .61** | *.34 / .35* | **.55 / .49** | **.51 /** *.46* | *.41 / .40* | **.72 / .67** | *.41 / .30* | -.21 / *-.30* | -.22 / -.15 |
| 1. CBCL Social problems (caregiver) | **.52 /** **.64** | *.49 /* **.68** |  | **.78 / .78** | *.39 / .43* | *.37 / .30* | **.71 / .59** | **.53 / .49** | **.70 / .67** | .30 / .18 | *-.35 / -.44* | *-.33 / -.34* |
| 1. CBCL Attention problems (caregiver) | **.72 / .83** | **.69 / .77** | **.56 / .62** |  | *.36 / .46* | *.36 / .31* | **.53 / .54** | **.63 / .56** | **.84 / .79** | **.47 /** *.34* | *-.30 / -.34* | **-.45 /** *-.34* |
| 1. TRF Internalizing (teacher) | .05 / .02 | -.13 / -.19 | -.05 / -.04 | -.08 / -.12 |  | **.48 / .66** | **.68 / .77** | **.48 / .59** | *.33 / .45* | *.38 / .45* | -.12 / -.01 | -.15 / -.02 |
| 1. TRF Externalizing (teacher) | -.03 / .15 | *.16* / .27 | .04 / .25 | .09 / *.39* | *.50 / .40* |  | **.58 / .72** | **.77 / .75** | **.59 / .58** | **.81 / .72** | .03 / -.08 | -.15 / -.01 |
| 1. TRF Social problems (teacher) | .15 / .04 | -.02 / -.27 | .14 / -.07 | -.04 / -.01 | **.70 / .84** | **.63** */ .55* |  | **.57 / .72** | **.51 / .57** | **.45 / .58** | -.23 / -.20 | -.23 / -.15 |
| 1. TRF Attention problems (teacher) | .15 / .17 | .16 / .03 | .18 / .09 | .25 / .35 | **.60 / .61** | **.78 / .71** | **.70 / .73** |  | **.77 / .78** | **.90 / .87** | -.17 / -.26 | *-.41 / -*.25 |
| 1. ADHD Rating Scale (caregiver) | *.44 / .44* | **.62 /** *.49* | *.42 /* *.46* | **.74 / .72** | -.02 / .05 | .31 **/ .57** | .11 / .20 | *.43 / .51* |  | **.64 / .60** | -.25 / -.26 | *-.40 / -*.24 |
| 1. ADHD Rating Scale (teacher) | .03 / .09 | .22 / .23 | .08 / .15 | *.26 / .40* | *.44 / .36* | **.79 / .79** | **.52 /** *.48* | **.90 / .83** | *.48 /* **.60** |  | -.07 / -.17 | *-.36 / -*.20 |
| 1. WISC-R: Total IQ score | .13 / -.06 | -.24 / -.15 | -.22 / -.15 | -.03 / -.10 | -.12 / -.10 | -.16 / -.25 | -.14 / -.11 | -.10 / -.19 | -.14 / -.26 | -.12 / -.12 |  | **.62 / .75** |
| 1. WISC-R: Freedom from distractibility | -.11 / -.15 | -.17 / -.04 | -.27 / -.05 | -.12 / .04 | *-.34* / -.32 | -.13 / .00 | -.22 / -.13 | -.11 / -.07 | .01 / .02 | -.01 / .20 | **.71 / .68** |  |

Note.

The figures are Spearman’s rho correlations / partial linear correlations for the drug-exposed group (top right) and comparison group (bottom left). Partial correlations are controlled for age at the time of assessment, gender, gestational age, birth weight and caregivers’ socioeconomic status. For comparability, only complete cases for all variables were included; thus, *n* = 48 in the drug-exposed group and *n* = 35 in the comparison group.

CBCL = Child Behavior Check List; TRF = Teacher Report Form.

*Italics* = *p* ≤ .05; **bold** = *p* ≤ .001.
